# Molecular systems biology at the crossroads: to know less about more, or to know more about less?

**DOI:** 10.1186/1477-5956-3-8

**Published:** 2005-10-28

**Authors:** Martin Latterich

**Affiliations:** 1 Department of Anatomy and Cell Biology, McGill University, Montreal, QC, H3A 2B2, Canada

## Abstract

Systems biology is a rapidly evolving discipline that endeavours to understand the detailed coordinated workings of entire organisms, with the ultimate goal to detect differences between health and disease, or to understand how cells or entire organisms react to the environment. The editorial provides a critical evaluation of what molecular systems analysis can and cannot accomplish with existing methodologies, and how systems biology needs to merge with reductionism to yield a more comprehensive and mechanistically insightful model of a cell or organism.

## Editorial

The recent few years have seen a growing interest in defining and establishing the emerging discipline of systems biology. While it is difficult to clearly define such a rapidly evolving discipline, characteristic trends are becoming apparent that allow a definition of what systems biology plans to accomplish. System biology endeavours to understand the detailed coordinated workings of entire organisms, with the ultimate goal to detect differences between health and disease, or to understand how cells or entire organisms react to the environment. Its ultimate goal is to understand the dynamic networks of regulation and interactions that allows cells and organisms to live in a highly interactive environment, and to understand how perturbations in the system cause disease.

Systems biology has been enabled by recent advances in multi-disciplinary scientific disciplines that allow for the parallel large-scale measurement of biomolecules, such as mRNA, proteins and metabolites. Understanding the detailed physiology of cells, tissues and entire organisms afforded by this approach will lead to a more comprehensive understanding of basic cellular events and their coordination. This comprehensive investigative approach represents a major shift in scientific paradigm, and over time will clearly have a major impact on how scientific analysis will be conducted.

The critics of systems biology are ready to point out that "omic" approaches are not a substitute for hypothesis driven research, because a systems analysis does not provide a testable hypothesis but is more akin a "fishing expedition", yielding minable information of a collective of molecules. However, this view-point does not do the discipline justice, because large scale investigative approaches can be hypothesis driven. For instance, one can form more global hypotheses such as a cell line or tissue changes protein expression/modification patterns in response to a drug stimulus, and that these changes are causally related to a toxic response to the drug. Using integrated molecular tools, these induced changes can readily be measured and compared to an appropriate experimental control. Cluster and correlation analysis of these data will then readily describe the dynamics of molecular changes in response to a perturbation of the system, in this case a drug challenge. Taken at face value, this collective information will provide the researcher with a foundation to create better-informed hypotheses. This then accelerates the discovery process by avoiding the sequential trial and error approach that often plague classical experimentation.

However, the inherent danger of current systems approaches lies in the temptation to over interpret the data and conclude predictions about mechanisms based on experimentally unproven correlations. For example, while it is easy to make pathway predictions by combining dynamic changes in cellular component concentration with prior knowledge about some (but not all) proteins, it is impossible to derive mechanistic insights from these data, because correlations alone cannot be considered scientific proof of mechanisms. Additionally, not all correlations are causally responsible for the phenotype of a cell or organism and can occur fortuitously.

The real question is what we wish to accomplish in modern biomedical research. Do we want to understand less about more, using a systems biology approach to understand global networks at the expense of mechanistic detail, or do we go on understanding more about less, using reductionist approaches aimed at understanding the mechanistic details of molecular machineries at the expense of comprehensive analysis. Both approaches clearly have their strengths and limitations, depending on what biological question needs to be answered. However, to fully understand the workings of a biological system in detail, both approaches need to be combined because they provide complementary data. The real issue is that high-throughput approaches, such as gene expression analysis, proteomics (the quantification and identification of proteins and their modifications), and metabolomics (the quantification of metabolites) provide only part of the cellular picture, namely the collective of molecules in a cell. When comparing the dynamic changes in molecular collectives between different experimental or environmental conditions, correlations become obvious that allow the generation of molecular or genetic networks of interdependence. While this information can provide great insights into how genetic and proteomic programs are modulated, the information alone does not provide any mechanistic details of how these molecules catalyze chemical reactions. The latter information can only be obtained through reductionist approaches, for example through the structural and functional analyses of proteins and the reconstitution of biological processes *in vitro*, which can scientifically prove function and mechanism. Knowledge about tissue specific and subcellular protein localization, together with quantitative information about local or cellular abundance, will add further detail that allows the interpretation and assessment of which machineries are localized where and if a given mechanism is likely to be significant to a particular process.

Experimental data from system analysis and reductionist approaches all have different formats. For example, vectorial protein interaction maps are different from reaction kinetic graphs. The key challenge in molecular systems biology is therefore how to integrate the data from high-throughput analyses with kinetic and mechanistic data obtained through reductionism, which is needed for the detailed description and modelling of cells and organisms. The challenge is not only a technical one. The real challenge lies in how to integrate both fields in a complementary fashion, and how to derive knowledge from datasets from vastly different disciplines in a meaningful manner. The latter will bring along new challenges to the bioinformatics community who will ultimately shift focus from pure data mining approaches to approaches that incorporate knowledge acquisition and determine cause-effect relationships between pathways and molecules. This experimental integration is more difficult, given the vastly different strategies taken by both disciplines. For the time being, high throughput approaches will yield a more global image of cellular dynamics, which in many cases can provide clues as to which pathway or molecules to focus on for further reductionist investigation.

Often, systems biology is viewed as the "next trend" after the genome project, suggesting it applies the knowledge contained within the genetic code to an organism's physiology. This statement implies that it might be as straight forward as sequencing the genome to describe a system. While the genome contains the blueprint of organisms and a program of how to properly develop into one, the description of the developmental and environmentally responsive programs has many more dimensions and requires us to understand the dynamics of how the collectives of molecules in a cell and organism interact and at what concentrations they are present in what specific localization. This brings along challenges of how to provide the measurements to acquire the necessary data, how to store and mine these data, and lastly how to efficiently derive mechanistic insights. The challenge to describe and model at the detailed systems level seems to be insurmountable from the technical perspective at this time. However, it is worthwhile remembering that when the human genome project firstly was proposed, many sceptics doubted that it would be technically feasible to sequence and assemble a genome as complex. With time and the appropriate resources spent, the next decade or two will undoubtedly see major advances in the modelling of complex systems, as well as in the creation of more mature technologies to detect, quantify, and functionally analyse cellular and organismal constituents. Looking even more into the future, it may well become possible to extend systems analysis to individuals, thus describing and perhaps predicting how such an individual may react to the environment or to medications, the latter of which is crucial for the development of personalized medicine. Individuals can respond to a given drug treatment regime in different ways, due to their distinct genetic make-up in addition to having experienced distinct environments that can coin a different physiology. It is therefore imperative to measure the molecular physiology prior and immediately after drug exposure to link dynamic changes in molecular composition to outcome therapeutic regimes or that of disease. Finding molecular signatures or actual biomarker that are predictive of disease or treatment outcome is a first step into that direction, where an individual systems analysis will ultimately help the care provider to customize a treatment regime.

## Competing interests

The author(s) declare that they have no competing interests.

## Authors' contributions

ML contributed 100 % to the Editorial Commentary.

